# Natural Marine Precursors Boost Continental New Particle Formation and Production of Cloud Condensation Nuclei

**DOI:** 10.1021/acs.est.4c01891

**Published:** 2024-06-13

**Authors:** Robin Wollesen de Jonge, Carlton Xavier, Tinja Olenius, Jonas Elm, Carl Svenhag, Noora Hyttinen, Lars Nieradzik, Nina Sarnela, Adam Kristensson, Tuukka Petäjä, Mikael Ehn, Pontus Roldin

**Affiliations:** † Department of Physics, Lund University, Professorsgatan 1, Lund SE-22363, Sweden; ‡ Swedish Meteorological and Hydrological Institute (SMHI), Norrköping SE-60176, Sweden; § Department of Chemistry, Aarhus University, Langelandsgade 140, Aarhus DK-8000, Denmark; ⊥ Finnish Meteorological Institute, Kuopio FI-70211, Finland; ∥ Department of Chemistry, Nanoscience Center, University of Jyväskylä, Jyväskylä FI-40014, Finland; # Department of Physical Geography and Ecosystem Science, Lund University, Lund SE-22362, Sweden; □ Institute for Atmospheric and Earth System Research/Physics, Faculty of Science, University of Helsinki, Helsinki FI-00014, Finland; ○ Joint International Research Laboratory of Atmospheric and Earth System Sciences, School of Atmospheric Sciences, Nanjing University, Nanjing CN-210023, China; △ Swedish Environmental Research Institute IVL, Malmö SE-21119, Sweden

**Keywords:** secondary aerosols, new particle formation, phytoplankton, dimethyl sulfide, modeling

## Abstract

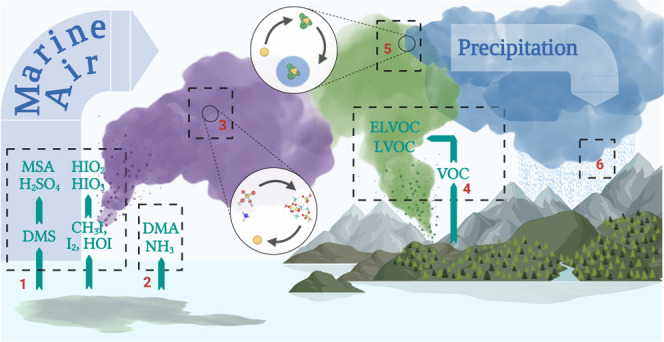

Marine dimethyl sulfide (DMS) emissions are the dominant source of natural sulfur in the atmosphere. DMS oxidizes to produce low-volatility acids that potentially nucleate to form particles that may grow into climatically important cloud condensation nuclei (CCN). In this work, we utilize the chemistry transport model ADCHEM to demonstrate that DMS emissions are likely to contribute to the majority of CCN during the biological active period (May-August) at three different forest stations in the Nordic countries. DMS increases CCN concentrations by forming nucleation and Aitken mode particles over the ocean and land, which eventually grow into the accumulation mode by condensation of low-volatility organic compounds from continental vegetation. Our findings provide a new understanding of the exchange of marine precursors between the ocean and land, highlighting their influence as one of the dominant sources of CCN particles over the boreal forest.

## Abstract 2

Little is known on the role of natural marine precursors in the formation of aerosol particles over land. Here we demonstrate that dimethyl sulfide in particular gives rise to a substantial fraction of aerosol particles in the cloud condensation nuclei size range observed over the Nordic boreal forest.

## 1 Introduction

Atmospheric aerosol particles play a critical role in controlling the Earth’s radiative balance. They absorb and scatter incoming solar radiation and govern the formation, radiative properties, and lifetime of clouds by acting as cloud condensation nuclei (CCN). Aerosols are produced naturally throughout various ecosystems and processes. In the oceans, phytoplankton blooms and bacteria provide the precursors necessary to form strong acids and bases capable of initiating the formation and growth of new particles from gases in the marine boundary layer.^1−3^ Important precursors include dimethyl sulfide (DMS: (CH_3_)_2_S) and iodine species CH_3_I, I_2_, and HOI, which oxidize in the atmosphere to produce sulfuric acid (SA: H_2_SO_4_), methanesulfonic acid (MSA: CH_3_SO_3_H), iodic acid (IA: HIO_3_), and iodous acid (HIO_2_), respectively.^4−8^ All acids are thought to nucleate, some among themselves and some in the presence of ammonia or alkylamines^9−13^ formed naturally in the surface ocean^2^ or emitted by anthropogenic sources. All acids along with ammonia and DMA continue to grow the newly formed particles from molecular clusters to CCN unless scavenged by larger particles.^3,14^

Over the continents, biogenic volatile organic compounds (BVOCs) are emitted from vegetation and oxidize in the atmosphere to produce low-volatility vapors that contribute to the growth of preexisting aerosol particles.^15,16^ Certain compounds of extremely low volatility (ELVOCs) also enable the growth of newly formed clusters.^17^ Monoterpenes (MTs) in particular (e.g., α-pinene) produce highly oxygenated organic molecules (HOMs) via the process of autoxidation.^18^ At least a fraction of these compounds are ELVOCs.^11^

Processes leading to the formation and growth of aerosol particles in both marine and continental environments have been studied extensively.^4,5,19,20^ Yet the interactions between marine and continental aerosols remain poorly understood. Other marine-continental exchanges are nevertheless described thoroughly in the literature. Water vapor is known to be transported from the oceans to the continents via the hydrological cycle, where it eventually condenses on available aerosol particles and leaves the atmosphere by precipitation. Therefore, it appears likely that marine precursors such as DMS, iodine species, and their oxidation products are also transported inland via air masses arriving from the oceans.

In the remote Arctic and Antarctic regions, IA, and DMS-derived SA along with ammonia and alkylamines are thought to be the main drivers of new particle formation (NPF) over land.^14,21−26^ Growth events are predominant in summertime during periods of high biological activity and occur when air masses arrive from the open ocean or retracting sea-ice regions.^23,25,26^

Over the boreal forest, NPF events correlate with elevated concentrations of MSA and alkylamines in the gas-phase and nucleation mode particles.^27−29^ The link between MSA, alkylamines, and NPF could suggest that precursors arriving from the marine environment affect aerosol processes over forested land. Various studies also report that particle formation and growth events are more frequent in the boreal forest when air masses arrive from the ocean.^29−32^ Lawler et al.^29^ argue that the effect is mainly caused by the low condensation sink conditions typical of air masses arriving from pristine marine regions, allowing for a larger fraction of the low volatile vapors to take part in NPF. They speculate, however, that precursors transported from the marine (e.g., DMS and iodine) in combination with the low condensation sink creates the optimal conditions for the formation and growth of new particles. While similar speculations have been presented in the literature, the impact of marine species such as DMS, iodine, NH_3_ and alkylamines on the formation and growth of aerosol particles over land has not been quantified.

In this work, we present model simulations of the gas-phase composition, NPF and secondary organic aerosol (SOA) growth over the boreal forest, with an emphasis on periods in which the air masses arrive from the marine environment. These simulations are able to estimate the scale to which marine precursors are transported inland from the ocean and quantify their effect on the formation and growth of aerosol particles in combination with condensable organic compounds produced over the forest. We use our model results to emphasize an until now poorly understood connection between the aerosol processes taking place over the forest and over the ocean and use this interaction to highlight the importance of marine precursors in the production of CCN sized aerosol particles over land. Finally, we demonstrate the influence of these marine-derived CCN sized particles on the formation of clouds and thereby the climate.

## 2 Materials and Methods

### ADCHEM Model Description

In order to verify the effect of marine precursors on the formation and growth of aerosol particles over the boreal forest, we utilize the chemical transport model ADCHEM to explicitly model the gas-phase chemistry, particle formation, and particle growth along the air-mass trajectories moving from the North Atlantic Ocean toward the boreal forest region. ADCHEM considers detailed aerosol dynamics including Brownian coagulation, wet and dry deposition along with condensation, dissolution, and evaporation of 873 organic and inorganic gas-phase species. The model comprises 20 vertical layers (spaced logarithmically) spanning 2100 m and considers mixing between all layers. Oxidation chemistry is treated in both the gas and aqueous phases, comprising a total of 5005 species and 13062 reactions. The chemical mechanism used includes MCMv3.3.1,^33,34^ the work on marine DMS oxidation by Wollesen de Jonge et al.^6^ and Hoffmann et al.,^5^ the work on iodine chemistry by Finkenzeller et al.,^8^ halogen chemistry from HM2.0 by Bräuer et al.^35^ and organic autoxidation from PRAM.^20^ The complete mechanism comprises the most advanced representation of the oxidation of DMS available in the current literature, including all potential reaction pathways in both the gas-phase and aqueous-phase. This includes the recently discovered compound hydroperoxymethyl thioformate (HPMTF), the formation of which was proposed in theory by Wu et al.,^36^ quantified experimentally by Berndt et al.^37^ and observed in the ambient atmosphere by Veres et al.^38^ The model also treats the aqueous-phase processing of 50 water-soluble species including DMS, methane sulphinic acid (MSIA), MSA, SA, SO_2_, O_3_, etc. in the deliquesced aerosol particle and cloud droplet, the latter of which was assumed to form at RH > 100% and activated at a constant cloud supersaturation of 0.3%.

Besides the base model setup (termed BaseCase), different sensitivity runs were performed to validate the effect of marine species on the formation and growth of aerosol particles over the boreal forest. An outline of the different sensitivity runs is given in Table 1. The first (named woDMS) represents a scenario without natural DMS emissions from the open ocean. The second (named woIodine) did not consider HIO_3_-HIO_2_ and HIO_3_-DMA clustering. The third (named woAnthro) did not include any anthropogenic particle and gas-phase emissions.

**Table 1:** Overview of the ADCHEM Model Base Case Setup and Sensitivity Runs

Model Run	Emissions	Chemistry	NPF
BaseCase	CAMS-GLOB-ANT	MCMv3.3.1 |HM2.0	SA-NH_3_ |SA-DMA
	CAMS-GLOB-BIO	PRAM |DMS^a^ |Iodine^b^	HIO_3_-HIO_2_ |HIO_3_-DMA
	CAMS-GLOB-OCE		
woDMS	Without emissions of DMS		
woIodine	Without HIO_3_–HIO_2_ and HIO_3_-DMA nucleation		
woAnthro	Without anthropogenic emissions of SO_2_, NO_*x*_, NH_3_, BC, CO, and AVOCs		

a DMS chemistry mechanism from Wollesen de Jonge et al.^6^

b Iodine chemistry from Finkenzeller et al.^8^

### Simulations Period, Location, and Measurement Data

ADCHEM was used to reproduce the gas and aqueous-phase chemistry along with particle formation and growth at the SMEARII field station in Hyytiälä, Finland (61.84°N, 24.28°E);^39,40^ Pallas field station, Finland (67.58° N, 24.07° E); and Hyltemossa field station, Sweden (56.06°N, 13.25°E), during 120 days in 2018 (first of May -28th of August). This period was chosen to match the occurrence of phytoplankton blooms forming in the North Atlantic and Arctic Ocean which govern the emission of DMS to the marine atmosphere. Due to the substantial data availability at SMEARII, the model performance was mainly evaluated against observation from said station. Modeled results from the Pallas and Hyltemossa stations can be found in the Supporting Information (see Figures S6 and S7). SMEARII nevertheless proved ideal to study the impact of DMS and other marine species in the aerosol dynamics taking place over the boreal forest, due to marine air masses arriving frequently at the station (see SI Figure S2). Model simulations of HOM monomers, SA, MSA, and HIO_3_ were validated against observations measured at 1.0 and 35.0 m by nitrate-ion-based chemical ionization atmospheric pressure-interface time-of-flight mass spectrometer (CI-APi-TOF). The instrument was calibrated for SA, with a calibration factor of 2.4 × 10^9^ molecules cm^–3^. The same calibration factor was used for HOM monomers, MSA and HIO_3_. Measurements of MTs and isoprene were obtained at 4.2 and 125 m by a proton-transfer-reaction mass spectrometer (PTR-MS) while particle number size distributions were measured by a differential mobility particle sizer (DMPS). ADCHEM was operated as an Lagrangian vertical column model^20,21^ along a total of 984 back-trajectories starting 7 days upwind from SMEARII, Pallas, and Hyltemossa and arriving at the field stations every third hour. All trajectories were generated by the Hybrid Single Particle Lagrangian Integrated Trajectory Model (HYSPLIT) and included meteorological data from the Global Data Assimilation System (GDAS).^41,42^

### Gas and Primary Particle Emissions

Daily emissions of marine BVOCs, DMS, and CH_3_I were obtained from the Copernicus Atmosphere Monitoring Service (CAMS) global ocean inventory at a 0.5° × 0.5° resolution.^43−46^ CAMS also provided monthly emissions of SO_2_, NH_3_, NO_*x*_, etc. from the global anthropogenic inventory at a 0.1° × 0.1° resolution.^47^ Emissions of continental BVOCs including isoprene along with MTs α-pinene, β-pinene, carene, and limonene were obtained from the CAMS global biogenic inventory.^48^ Emissions of isoprene over the boreal forest were scaled by a factor five in accordance with emissions simulations made by the global dynamic vegetation model LPJ-GUESS (Smith et al.;^49^ Arneth et al.^50^), which estimated isoprene emissions to be significantly higher than the ones presented in the CAMS-GLOB-BIO database. The ocean-atmosphere exchange of NH_3_ was calculated in accordance with the method described by Carpenter et al.^2^ taking into account the dependence of p*K*_a_ for the NH_3_–NH_4_^+^ equilibrium on sea surface temperature and salinity. Sea surface concentrations of NH_4_^+^ in the North Atlantic Ocean were estimated using results from Paulot et al.^51^ Emissions of DMA were treated similarly assuming a similar sea surface temperature and salinity dependence. Due to the uncertainty in the sea surface concentrations of NH_4_^+^ and DMA, natural emissions of NH_3_ and DMA are likewise uncertain. In addition to the emissions of organic iodine species treated in CAMS, inorganic iodine emissions of I_2_ and HOI where treated in accordance with the study by Carpenter et al.^52^ Sea spray was implemented in accordance with the parametrization by Sofiev et al.^53^ taking into account the 10 m wind speed and the sea surface temperature. The organic mass fraction of the sea spray aerosol was derived from the sea surface chlorophyll-a concentration as demonstrated by Gantt et al.^54^

### New Particle Formation

In this work, we simulated new particle formation by employing a molecular cluster plugin (ClusterIn) that simulates gas-cluster-aerosol interactions and feedbacks by explicitly coupling the cluster and aerosol dynamics.^55^ This method enables explicit temporal simulation of the concentrations of clusters and gas-phase clustering species, and coagulation between clusters and aerosols that constitutes a sink for clusters and a growth process for aerosols, in addition to growth by vapor condensation. This approach circumvents artifacts that may arise from commonly applied simplifications in particle formation dynamics, thereby enabling improved assessment of the importance of given chemical pathways for particle formation.^55^ To assess the role of different NPF mechanisms, namely ion-induced and neutral SA-NH_3_ and, SA-DMA, neutral HIO_3_-HIO_2_, and neutral HIO_3_-DMA, we employ the most recent molecular thermochemistry data sets as input for ClusterIn. The SA-NH_3_ and SA-DMA data sets are both computed at the DLPNO-CCSD(T)/aug-cc-pVTZ//ωB97X-D/6-31++G(d,p) quantum chemical level of theory, while the HIO_3_-HIO_2_ data set applies the DLPNO–CCSD(T)/M06–2 × 7/def2-TZVp level, and the HIO_3_-DMA data set applies the RI-CC2/aug-cc-pVTZ-PP//ωB97XD/6-311++G(3df,3pd) level.^56−59^ Steady-state particle formation rates simulated using the thermochemical data sets SA-NH_3_ and HIO_3_-HIO_2_ have previously been compared to experimental data from the CLOUD chamber and have agreed well with measurements performed at low temperatures and moderate-to-high base concentrations (for NH_3_ and the SA-NH_3_ data).^56,57^ This good agreement with CLOUD measurements can be expected to be due to the application of the state-of-the art DLPNO method for single-point energy calculations,^60,61^ and the quasi-harmonic corrections employed in the calculations which generally reduce cluster overbinding.^57^ Myllys et al.^58^ compared formation rates based on the SA-DMA quantum chemical data set and found good agreement with the CLOUD experiments at both low and high DMA gas concentrations. One should note that since the HIO_3_-DMA cluster thermochemical data involves single-point energy calculations by the RICC2 method that has a tendency toward overprediction of cluster stabilities and thereby formation rates, quantitative assessments of the relative importance or contribution of HIO_3_-HIO_2_ (based on DLPNO) and HIO_3_-DMA to iodine-driven particle formation cannot be performed. This implies that the HIO_3_-DMA particle formation rates should be considered to be an upper limit to the real NPF rate.

### Henry’s Law Solubility and p*K*_a_

Henry’s law solubility and p*K*_a_ of MSIA, MSA, HIO_3_, and HIO_2_ in water were estimated using the conductor-like screening model for real solvents (COSMO-RS^62−64^) implemented in the COSMO*therm*21 program.^65^ COSMO*therm* was used in order to describe the dependency of the Henry’s law solubility and p*K*_a_ values on temperature, thus providing the ADCHEM model with a more realistic representation of the uptake and dissolution of these water-soluble species under different atmospheric conditions. These calculations combine density functional theory calculations at the BP/def2-TZVPD-FINE//BP/def-TZVP level of theory^66,67^ and statistical thermodynamics using the most recent parametrization of COSMO*therm*, BP_TZVPD_FINE_21. Both properties were computed for temperatures from 250 to 300 K with 10 K intervals.

Henry’s law solubility is calculated from the activity coefficient of the compound in the infinite dilution in the solvent γ^∞^ saturation vapor pressure of the pure compound *p*_sat_, as well as the density (ρ) and molar mass (*M*) of the solvent water:
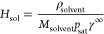
1The p*K*_a_ of a compound is estimated by using the free energy (*G*) of the neutral and ionic species at infinite dilution with the linear free energy relationship (LFER):
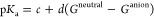
2The LFER parameters of the BP_TZVPD_FINE_21 parametrization for water as a solvent are *c* = −130.024 and *d* = 0.486 mol kcal^–1^. It should be noted that the p*K*_a_ calculation in COSMO*therm* is parametrized only for room temperature (around 300 K).

### Adiabatic Cloud Parcel Model

The amount of activated cloud droplets was estimated by using an adiabatic cloud parcel model.^68,69^ Calculations were done at two different up-draft velocities, w = 0.1 m/s and w = 1.0 m/s, using the size distribution, size resolved chemical composition, and gas-phase concentration of SO_2_, H_2_O_2_, NH_3_, HNO_3_, SA, and HCl obtained from the ADCHEM model *BaseCase*, *woDMS*, *woIodine*, and *woAnthro* runs as input. The air parcel was assumed to rise from ground-level at a relative humidity (RH) of 98%, ultimately reaching 160 m of altitude. Upon reaching supersaturation (RH > 100%) with respect to water, aerosol particles in the CCN size range would activate into cloud droplets. The time by which supersaturation was reached in the air parcel depended on the updraft velocity.

### Air-Mass Origin Analysis

Particle number size distributions obtained by DMPS measurements from SMEARII, Pallas, and Hyltemossa were grouped into percentiles based on the amount of time that their corresponding air masses spend over the ocean before reaching each station. The separation was done for observations obtained between 2006 and 2020 at SMEARII, 2015–2018 at Pallas and 2018–2020 at Hyltemossa, considering only spring and summertime data in order to represent the peak period in north Atlantic and Arctic ocean marine phytoplankton activity and thus DMS emission. Furthermore, only daytime observations between 6.00 and 18.00 representative of local NPF and growth at the stations were used. HYSPLIT back-trajectories were calculated at one h intervals starting 7 days upwind from each station, comprising in total 24655, 6570, and 4932 individual air masses arriving at SMEARII, Pallas, and Hyltemossa, respectively. Marine impact in terms of time spend over the ocean was obtained for each back-trajectory (see SI Figure S3) and used to classify the corresponding particle number size distribution.

### Statistical Methods

Model and observational means, medians, and percentiles were calculated using the built in functions *mean*, *median*, and *prctile* provided by MATLABR2022a. Correlation coefficients *R* were obtained by the *fitlm* function, while the normalized mean bias (NMB) and the fraction of model predictions (M) within a factor of 2 from observations (O) (FAC2) were calculated using eq 3 and eq 4, respectively.
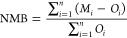
3
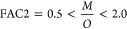
4

## 3 Results and Discussion

### Air Mass History and Aerosol Size Distributions

**Figure 1:**
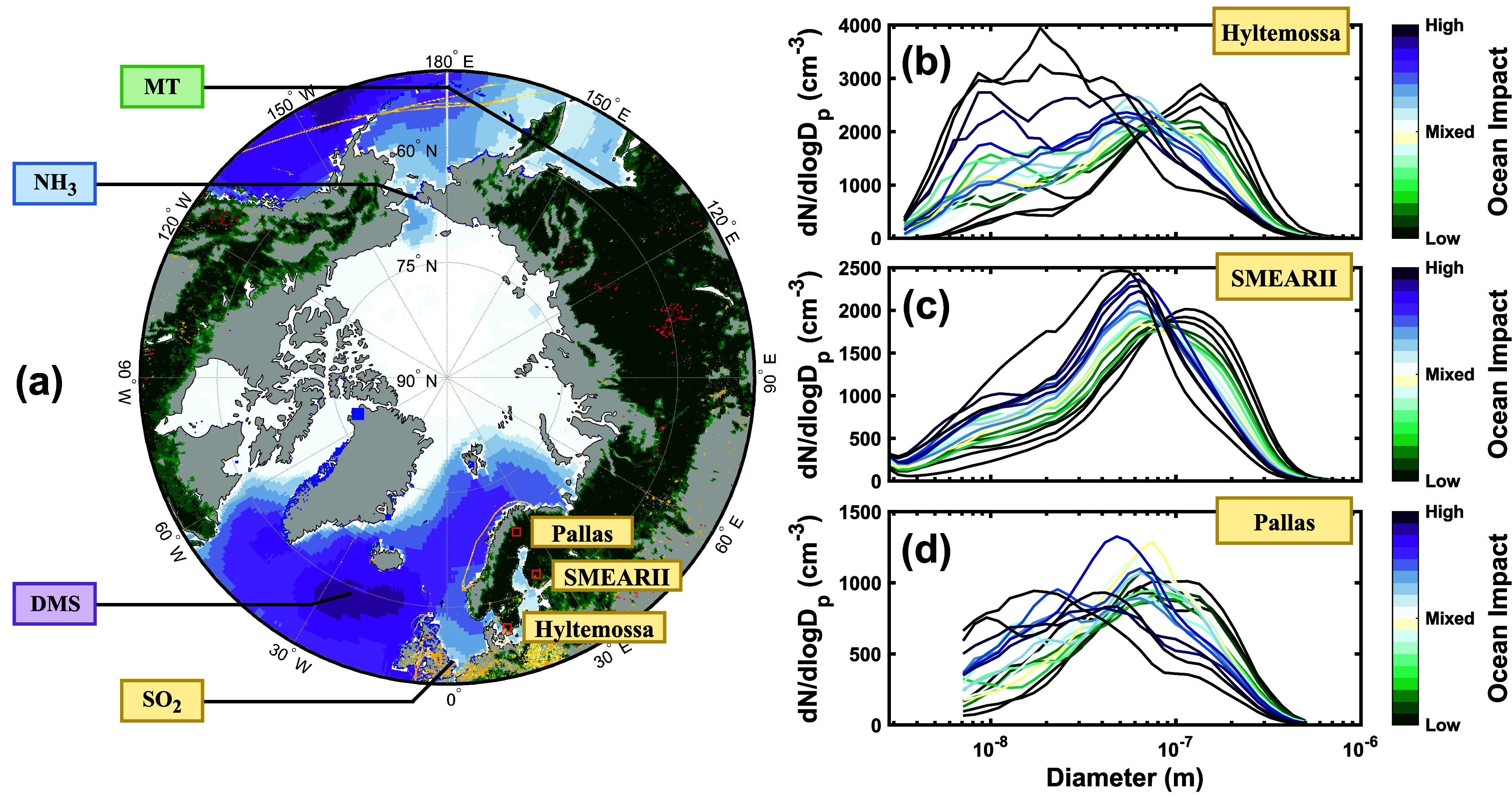
Effect of marine versus continental air mass history on the aerosol number size distribution in a boreal forest environment. Panel (a) depicts the typical summertime source regions of DMS (purple), MTs (green), NH_3_ (blue), and anthropogenic SO_2_ (yellow), along with their position in relation to the Hyltemossa, SMEARII, and Pallas measurements stations. These colors indicate only the spatial locations of the emissions of the different species between May and August, 2018. Panels (b), (c), and (d) show the spring and summertime (March-August) mean aerosol number size distributions measured at the Hyltemossa, SMEARII, and Pallas stations during the periods 2018–2020, 2006–2020, and 2015–2018, respectively. The size distributions in panel (b), (c,) and (d) are separated based on the marine influence on their air mass history.

Air mass origin has a profound impact on the gas-phase chemistry, new particle formation, and particle growth over the boreal forest. Various regions emit different types of primary particles and precursor gases, all of which are mixed and moved around by large-scale weather systems. Figure 1a illustrates the regions that are particularly prone to affect aerosol processes at the Hyltemossa (56°06′N, 13°25′E), SMEARII (61°51′N, 24°17′E), and Pallas (67°58′ N, 24°07′ E) research stations, of which SMEARII and Pallas are located in the Finnish boreal forest and Hyltemossa in the southern Swedish temperate forest. Clean air masses from the North Atlantic Ocean are known to transport DMS, sea spray and NH_3_ emitted from natural processes in the surface ocean, while air masses from the southwest and southeast are dominated by anthropogenic emissions of SO_2_, NH_3_, NO_*x*_ and black carbon (BC). Natural emissions of biogenic volatile organic compounds (BVOCs) such as MTs and isoprene influence the aerosol processes close to the stations.

The effect of different air mass origins on the aerosol processes over the boreal forest becomes evident when separating the observed aerosol number size distributions at the Hyltemossa, SMEARII, and Pallas research stations based on the influence of air masses from different source regions. Figure 1b, 1c and 1d depicts the mean aerosol number size distribution during spring and summertime at the Hyltemossa, SMEARII and Pallas stations during years 2018–2020, 2006–2020, and 2015–2018, respectively. Each size distribution is grouped and colored based on the amount of time that its associated air-mass back-trajectory spent over the ocean before reaching the station (see Methods). Consequently, the purple size distributions are dominated by air masses arriving from the marine, while the green size distributions are dominated by air masses arriving from the continent. All stations show a tendency toward a higher particle number concentration in the nucleation and Aitken mode particle size range (PN_<25 nm_ and PN_25–100 nm_, respectively) when marine regions dominate the air mass history, while air masses with continental influence are shifted toward the accumulation mode size range (PN_100–1000 nm_). PN_<25 nm_ at Hyltemossa, SMEARII and Pallas are 14.7, 5.3, and 9.3 times higher, respectively, for size distributions with an marine air-mass influence above the 90th percentile compared to size distributions with an marine air-mass influence below the 10th percentile. PN_25–100 nm_ are correspondingly 2.7, 2.2, and 1.6 higher for periods of high versus low marine influence while PN_100–1000 nm_ is 0.29, 0.36, and 0.34 times as high. This result suggests that marine air masses favor the formation of particles in the nucleation mode size range along with their subsequent growth into the Aitken mode size range over the boreal forest. A possible explanation may be the low condensation sink associated with the clean air masses arriving from the North Atlantic Ocean. This would inevitable lower the loss of locally emitted anthropogenic SO_2_-derived SA and NH_3_ to the accumulation and coarse mode particles and allow these gases to contribute to NPF instead. At the same time, DMS-derived SA, HIO_3_, NH_3_ and DMA naturally emitted from the ocean may also contribute to NPF upwind from and over the boreal forest, thereby adding to the nucleation and Aitken mode particle number concentration observed at the SMEARII, Hyltemossa and Pallas stations.

In conclusion, separating multiple years of aerosol number size distributions measured at three boreal forest stations based on the influence of marine air masses shows an increase in the nucleation mode and Aitken mode particle number concentration for air masses having spent most of their time over the ocean.

### Marine Air-Mass Influence on the Gas-Phase Chemistry over the Boreal Forest

Anthropogenic SA and NH_3_ along with HOM produced from the oxidation of BVOCs are often thought to be the main drivers behind the formation and growth of aerosol particles over the boreal forest. Size distributions separated by marine impact on the other hand suggests that marine species and their oxidation products may promote the nucleation and Aitken mode particle production during periods of high marine air-mass impact. We assess the impact of marine species in the gas and particle phase over the boreal forest using the ADCHEM model and evaluate the performance of ADCHEM in reproducing the precursors, oxidation species, and oxidation products that govern the aerosol processes at the SMEARII, Pallas and Hyltemossa stations (see the Supporting Information).

**Figure 2:**
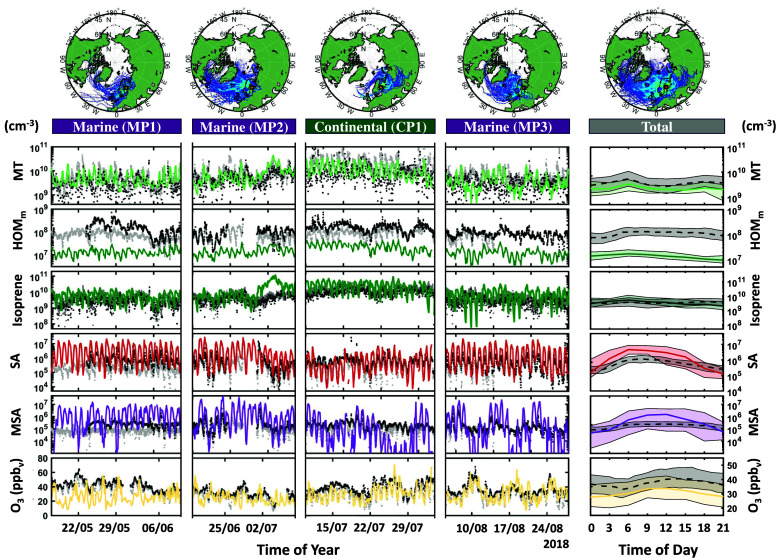
Modeled and measured long-term and diurnal gas-phase concentrations of MTs, HOM monomers, isoprene, SA, MSA, and O_3_ at the Station for Measuring Ecosystem-Atmosphere Relations II (SMEARII) between the 17th of May and 28th of August, 2018. Gray and black dots denote the surface and above-canopy measurements, respectively, while the colored lines represent the above-canopy model results. Marine periods 1, 2, and 3 (MP1, MP2, and MP3) denote periods of particularly high marine air-mass impact, while continental period 1 (CP1) denotes a period of particularly high continental air-mass impact. The back-trajectory heat-maps displayed above each period illustrate the regions from which the air-masses arrived during MP1, MP2, MP3, and CP1. The shaded areas denote the measured and modeled data range within the 25th and 75th percentile.

Figure 2 compares the modeled and measured long-term and diurnal gas-phase concentrations of MTs, HOM monomers, isoprene, SA, MSA, and O_3_ at the SMEARII station during four distinct periods spanning from the 17th of May to the 28th of August, 2018. Marine period one, two and three (MP1:17/5–10/6, MP2:19/6–8/7 and MP3:5/8–28/8) denotes periods of high marine air-mass impact, while continental period one (CP1:10/7–3/8) denotes a period of high continental air-mass impact. Table 2 summarizes the modeled and measured median gas-phase concentrations, the correlation coefficient (R), the normalized mean bias (NMB) and the fraction of model predictions within a factor of 2 of the measurements (FAC2). Additional concentrations of modeled gas-phase species can be found in the SI (see SI Figure S1). Considering the measurement uncertainty of HOMs, SA and MSA observations obtained by HR-ToF-CIMS, ADCHEM is able to reproduce gas-phase species at the SMEARII station. SA and MSA, while overestimated in the model (NMB(SA) = 2.19, NMB(MSA) = 5.69) show relatively low but significant correlation for the long-term time series (*R*(SA) = 0.24, *R*(MSA) = 0.29) and relatively high correlation for the diurnal cycle (*R*(SA) = 0.96, *R*(MSA) = 0.84). The modeled MSA gas-phase concentration does nevertheless undergo higher variability compared to the measured concentration, which could indicate that MSA is more volatile than currently predicted by COSMO*therm* calculations in the model.^6,70^ BVOCs isoprene and MTs show relatively low although significant correlation with measurements for the whole time period (*R*(Iso) = 0.36, *R*(MT) = 0.32), and are both within a factor of 2 from the observations for 45% of the time. The discrepancy between model and measurements appears to arise from the diurnal cycle, which may be captured less well by the model due to the simplified representation of the boundary layer dynamics. HOM monomers are consistently underestimated by the model (NMB(HOM_*m*_) = −0,89), with a relatively low but significant correlation for the total time series (*R*(HOM_*m*_) = 0.39) and the diurnal cycle (*R*(HOM_*m*_) = 0.23). We speculate if the discrepancy between model and measurement is caused by different definitions of HOM. While ADCHEM only classifies molecules produced via MT autoxidation as HOM, the estimated HOM concentrations from the HR-ToF-CIMS include all detected molecules with six or more oxygen atoms. Ozone, which plays a key part in the oxidation of MTs and DMS, shows relatively high correlation for the diurnal cycle (*R*(O_3_) = 0.61) and stays within a factor of 2 from the observations for 86% of the time. While the long-term ozone correlation is relatively low (*R*(O_3_) = 0.39), the low normalized mean bias (NMB(O_3_) = −0.03) suggests that the absolute ozone concentration is reproduced by the model.

**Table 2:** Evaluation of the Modeled Gas-Phase Concentration of MTs, HOM Monomers (HOM_*m*_), Isoprene, SA, MSA, and O_3_ at the Station for Measuring Ecosystem-Atmosphere Relations II (SMEARII)^a^

Species	*O̅* (cm^–3^)^b^	*M̅* (cm^–3^)	*R*	NMB	FAC2
MT	3.74 × 10^9^	2.20 × 10^9^	0.32 (0.81)	–0.48 (−0.38)	0.45 (1.00)
HOM_*m*_	1.09 × 10^8^	1.37 × 10^7^	0.39 (0.23)	–0.87 (−0.87)	0.03 (0.00)
Isoprene	3.31 × 10^9^	3.49 × 10^9^	0.36 (0.38)	0.31 (−0.01)	0.45 (1.0)
SA	5.25 × 10^5^	1.10 × 10^6^	0.24 (0.96)	2.19 (2.19)	0.23 (0.25)
MSA	1.82 × 10^5^	1.80 × 10^5^	0.29 (0.84)	5.69 (2.17)	0.17 (0.50)
O_3_	32.5 (ppb)	30.66 (ppb)	0.39 (0.61)	–0.03 (−0.18)	0.86 (1.00)

a A comparison between the modeled and measured concentrations is provided for the full period between May and August and for the diurnal variability in the gas-phase concentrations averaged over the entire period.

b *O̅*denotes the observed median gas-phase concentration of each species,*M̅* the modeled median gas-phase concentration, *R* the correlation coefficient, NMB the normalized mean bias, and FAC2 the fraction of predictions within a factor two of the observations. Diurnal results are reported in parentheses.

DMS constitutes a significant source of SA at the SMEARII station. Comparing model results from the *BaseCase* setup with that of the *woDMS* sensitivity run suggests that DMS contributes to 65% of the gas-phase and particle-phase SA observed at the station for the total simulation period. The fraction of DMS-derived SA increases to 66%, 79%, and 68% during MP1, MP2, and MP3, respectively. While the majority of SA related to the oxidation of DMS resides in the particle-phase upon reaching the SMEARII station, DMS-derived SO_2_ ensures a steady production of gas-phase SA over the boreal forest due to its prolonged lifetime. Consequently, DMS provides 47% of the gas-phase SO_2_ observed at the station for the entire simulation period along with 63%, 71%, and 44% during MP1, MP2, and MP3, respectively. The inland transport of DMS and its associated oxidation products is also evident from the *woAnthro* sensitivity run. Running the model without any emissions of anthropogenic sulfurous compounds suggests that DMS alone is able to maintain a mean SO_2_ gas-phase concentrations at the SMEARII station of 3.3 × 10^9^ cm^–3^, 3.7 × 10^9^ cm^–3^, and 2.1 × 10^9^ cm^–3^ during MP1, MP2, and MP3, respectively. The consequent SA gas-phase concentration was found to be 5.1 × 10^6^ cm^–3^ during MP1, 3.7 × 10^6^ cm^–3^ during MP2, and 2.2 × 10^6^ cm^–3^ during MP3.

These results suggests that DMS constitutes an important if not dominant source of SO_2_ and SA to the present day spring and summertime Scandinavian boreal forest atmosphere, but also that DMS would be likely to ensure significant SO_2_ and SA gas-phase concentrations in said forest without anthropogenic influence. Results from the *woAnthro* sensitivity run also indicate that the mean NH_3_ concentrations may reach 2.2 × 10^8^ cm^–3^, 9.1 × 10^8^ cm^–3^ and 2.2,·10^9^ cm^–3^ during MP1, MP2 and MP3, respectively, from natural marine sources alone. While this is but a fraction of the NH_3_ gas-phase concentration caused by anthropogenic sources, it does provide the amount necessary to initiate NPF over the boreal forest in combination with SA derived from DMS under certain favorable conditions.

The inland transport of iodine species CH_3_I, I_2_, and HOI produces an HIO_3_ concentration at the SMEARII station of 3.3 × 10^5^ cm^–3^, 3.3 × 10^5^ cm^–3^, 6.8 × 10^5^ cm^–3^, and 2.1 × 10^6^ cm^–3^ during MP1, MP2, MP3, and CP1, respectively (see SI Figure S1b). The increased HIO_3_ concentration during CP1 coincides with the continental dominated air masses spending a significant fraction of time over coastal areas (see Figure S2), most of which are rich in kelp and algae, which favors the emission of CH_3_I.

BVOCs (isoprene and MTs) along with their associated oxidation products are dominant during periods of high continental impact. Consequently the gas-phase and particle-phase concentration of all condensable HOM species is 1.8, 1.7, and 2.0 times higher during CP1, compared to MP1, MP2 and MP3, respectively. The production of low-volatility organic compounds nevertheless remains high during periods of high marine impact, with the potential to grow newly formed particles from both natural and anthropogenic SA and NH_3_ into larger aerosol particles.

Overall, the model results together with the observations at SMEARII indicate that DMS has a profound impact on the gas-phase and particle-phase concentration of SA and SO_2_ over the boreal forest during periods of high marine air-mass impact and high biological activity. Furthermore, sensitivity runs indicate that natural marine species alone are capable of providing significant concentrations of SA, IA, and NH_3_ at the SMEARII station without any impact from anthropogenic sources.

### Marine Air-Mass Impact on Particle Formation and Growth over the Boreal Forest

**Figure 3:**
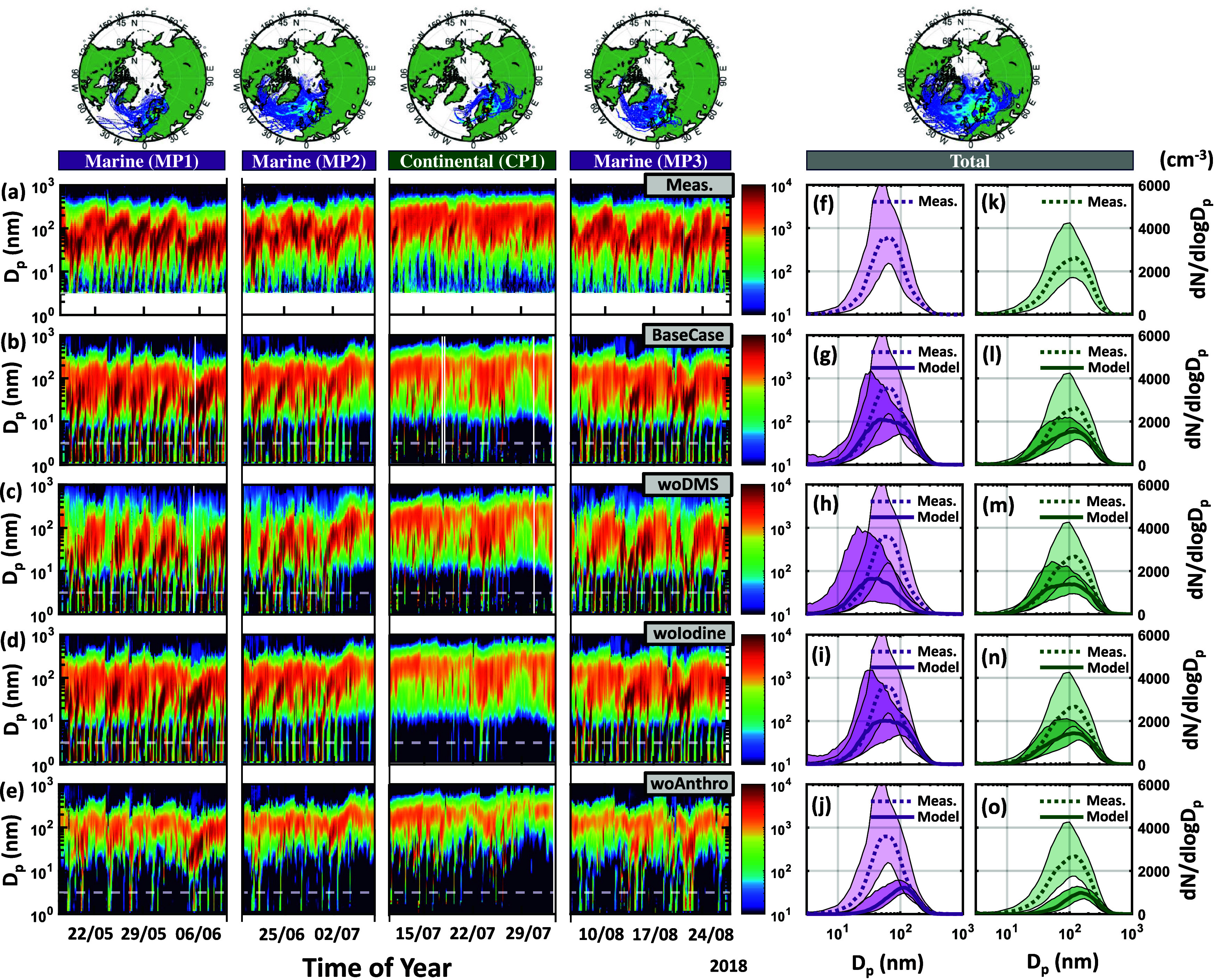
Measured and modeled (a–e) time-dependent and (f–o) median particle number size distributions at the Station for Measuring Ecosystem Atmosphere Relations II (SMEARII) between the 17th of May and 28th of August. Marine periods 1, 2, and 3 (MP1, MP2, and MP3) denote periods of particular high marine air-mass impact, while continental period 1 (CP1) denotes a period of particular high continental air-mass impact. The back-trajectory heat-maps displayed above each period illustrates the regions from which the air-masses arrived during MP1, MP2, MP3, and CP1. The median size distributions are separated into (f–j) periods of predominant marine air-mass impact (time spent over the ocean >50th prct., purple), and (k–o) periods of predominant continental impact (time spent over the ocean <50th prct., green). The averaging for the median size distributions is done for the whole simulation period and not just during MP1, MP2, MP3, and CP1. The model results include data from the base case run (BaseCase), the without DMS emissions simulation (woDMS), the without iodine nucleation simulation (woIodine), and the without anthropogenic emissions simulation (woAnthro). The shaded areas denote the measured and modeled data range within the 25th and 75th percentile.

Considering the influence of marine species on the gas-phase concentration of SO_2_, SA, HIO_3_, and NH_3_ both at and upwind from the SMEARII station, air masses arriving from the ocean are likely to affect the formation and growth of aerosol particles over the boreal forest. Figure 3 compares the measured and modeled particle number size distributions during MP1, MP2, MP3, and CP1, along with median size distributions separated into periods dominated by marine or continental air-mass history. Model results without the influence of DMS (Figure 3c), without the influence of iodine-derived NPF (3d), and without the influence of anthropogenic precursors (Figure 3e) are illustrated by the *woDMS*, *woIodine*, and *woAnthro* sensitivity runs. Considering the *BaseCase* model setup, ADCHEM predicts the trends in particle formation and growth at the SMEARII station. The modeled median size distribution during periods of predominant marine air-mass impact (time spent over the ocean >50th prct) shows relativly high correlation with measurements (*R* = 0.96) with a small underestimate in the absolute particle number concentration (NMB = −0.24) (Figure 3g). Periods of low marine air-mass impact (time spent over the ocean <50th prct.) also shows relatively high correlation with observations (*R* = 0.98) but underestimates the absolute particle number concentration to a larger extent (NMB = −0.33) (Figure 3l). Similar results for the Pallas and Hyltemossa stations are provided in the Supporting Information (see Figure S6 and Figure S7, respectively).

ADCHEM is able to reproduce the increase in nucleation and Aitken mode particle production during periods of high marine air-mass impact as presented in previous sections. Consequently, the median particle number concentration is 2.8 times higher in the nucleation mode and 1.7 times higher in the Aitken mode during periods of high marine impact (>50th prct.) as opposed to periods of low marine impact (<50th prct.), respectively (Figure 3g,l). The increase in the number of nucleation and Aitken mode particles may to some extent be explained by the low condensation sink associated with air masses arriving from the ocean. During CP1 the average modeled condensation sink was found to be 4.0 × 10^–3^ s^–1^ as opposed to 2.8 × 10^–3^ s^–1^, 3.2 × 10^–3^ s^–1^, and 2.7 × 10^–3^ s^–1^ during MP1, MP2 and MP3, respectively. However, according to the *woDMS* and *woIodine* sensitivity run the nucleation and Aitken mode particle production cannot be explained by the combination of low condensation sink and anthropogenic sources of SA and NH_3_ alone. Running the model without DMS emissions decreases the Aitken mode (PN_25–100nm_) and accumulation mode (PN_>100nm_) particle number concentration by 12% and 42% for the entire simulation period and by 26% and 68% during periods of predominant marine air-mass impact (time spent over the ocean >50th prct.), respectively (Figure 3h). At the Pallas and Hyltemossa stations the particle number concentrations across all particle sizes are reduced by 71% and 25% for the entire simulation period, respectively (Figures S6 and S7). These results show the importance of DMS in the formation and growth of aerosol particles both locally and upwind from the SMEARII, Pallas and Hyltemossa research stations and implies that DMS plays a much larger role over the boreal forest than previously anticipated. Consequently, the low condensation sink associated with marine air masses make up ideal conditions for DMS-derived NPF via SA, DMA, and NH_3_ over the oceans and land, which eventually act as seed particles for condensational growth of organics over the forest. The effect of running the model without DMS at the SMEARII station is particularly prominent during the fourth, fifth and sixth of June where the nucleation, Aitken and accumulation mode is close to nonexistent in the model, and similar cases are seen consistently throughout MP1, MP2, and MP3 (Figure 3c). This would suggest that DMS not only contributes sporadically to the formation of aerosol particles over the boreal forest but also dominates the NPF and growth during certain periods of strong marine air-mass impact.

Iodine constitutes a smaller but nevertheless noticeable effect on the formation of particles at the SMEARII station. Running the model without the HIO_3_-HIO_2_ and HIO_3_-DMA nucleation mechanisms decreases the nucleation mode (PN_<25nm_) and Aitken mode (PN_25–100nm_) particle number concentration by 7% and 2% during periods of predominant marine air-mass impact (time spent over the ocean >50th prct.) and by 13% and 8% during periods of predominant continental air-mass impact (time spent over the ocean <50th prct.), respectively (Figure 3i,n). The effect of HIO_3_–HIO_2_ and HIO_3_-DMA nucleation on the accumulation mode (PN_>100nm_) particle number concentration remains negligible at the SMEARII station. The influence of iodine in the formation of nucleation and Aitken mode particles during periods of predominant continental air-mass impact corresponds with the air masses spending less time over the open ocean and more time over coastal areas (see SI Figure S2). These areas are rich in emissions of organic iodine species such as CH_3_I, emitted from kelp or algea, and adds a significant source of HIO_3_ and HIO_2_ during a period otherwise dominated by VOC emissions over the continent. The effect is evident during CP1, where small bursts in nucleation and Aitken mode particles are observed in the *BaseCase* simulation but not in the *woIodine* sensitivity run (Figure 3d).

The influence of DMS and iodine in combination with the natural emissions of NH_3_ and DMA is also illustrated by the *woAnthro* sensitivity run (Figure 3d). Running the model without anthropogenic emissions of SO_2_, NH_3_, NO_*x*_, etc., suggests that marine species alone are capable of forming new particles over the ocean and land, while emissions of BVOCs and their associated oxidation products grow said particles into the accumulation mode and potential CCN size range. Although this scenario is nonrepresentative of the present day polluted atmosphere over the boreal forest, it provides an insight to the role of marine species in the NPF and thus ultimately CCN production over land during preindustrial times.

Overall, modeling the influence of marine species on the aerosol processes taking place over the boreal forest suggests that DMS and iodine have a substantial impact on the formation and growth of aerosol particles both locally and upwind from the SMEARII, Pallas, and Hyltemossa stations during the biological active period of phytoplankton in the north Atlantic and Arctic ocean. Furthermore, DMS in particular not only adds sporadically to the nucleation, Aitken, and accumulation mode particle number concentration but dominates the particle production in said modes during periods of high marine air-mass impact.

### Impact of Marine Species on Clouds and Climate Over the Boreal Forest

**Figure 4:**
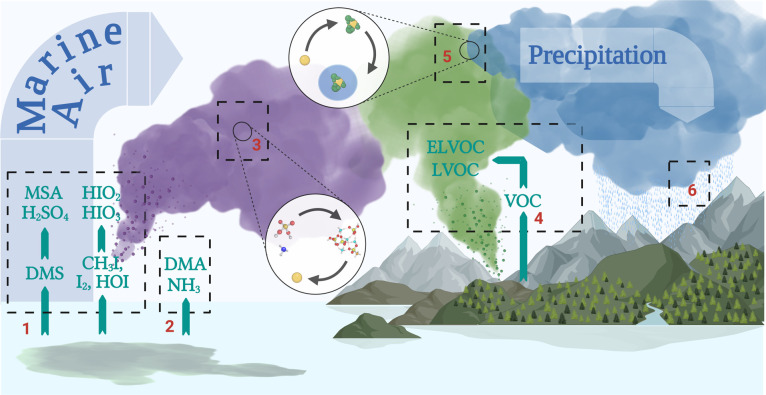
Schematic depiction of the influence of DMS and various iodine species on the formation and growth of aerosol particles both over the ocean and over land. (1) DMS, CH_3_I, I_2_, and HOI oxidize over the ocean and land to form the strong acids SA, MSA, HIO_3_, and HIO_2_, which nucleate (3) among themselves or (2) in the presence of NH_3_ or DMA. These particles in turn are (4) grown by condensation of low volatile organic compounds over the boreal forest, ultimately reaching (5) the CCN size range, where they (6) affect the formation, lifetime, and precipitation of clouds. Created with BioRender.com.

The contribution of DMS and iodine to the formation, lifetime, and precipitation of clouds and consequently climate depends on their ability to produce aerosol particles in the CCN size range. Figure 4 illustrates the process by which DMS and iodine may do so. In order to quantify the production of CCN from DMS and iodine, the modeled particle number size distribution, size resolved chemical composition, and gas-phase concentrations at the SMEARII station were used as input to an adiabatic cloud parcel model.^68,69^ Using this model, we calculated the amount of aerosol particles that activated into cloud droplets at various updraft velocities (*w*) (see Materials and Methods).

Table 3 summarizes the change in the CCN particle number concentration at updraft velocities *w* = 0.1 ms^–1^ and *w* = 1.0 ms^–1^ due to running the ADCHEM model without emissions of DMS, without iodine-derived NPF and without anthroprogenic emissions. According to the model, DMS has a substantial effect on the formation of CCN particles at both updraft velocities. Running the model without emissions of DMS decreases the number concentration of CCN by 78%, 82%, and 69% at *w* = 0.1 ms^–1^ and by 61%, 39%, and 51% at *w* = 1.0 ms^–1^ during MP1, MP2, and MP3, respectively. Updraft velocities in the range of 1.0 ms^–1^ are typical for the formation of cumulus clouds, whereas an updraft velocity of 0.5 ms^–1^ and below are more often seen in stratiform clouds.^71^ In accordance with the adiabatic cloud model, DMS constitutes a large source of CCN particles in both types of clouds and thus both ends of the CCN size range. This coincides with the median minimum dry diameters of the particles that activate into cloud droplets being 167 nm at 0.1 ms^–1^ and only 75 nm at 1.0 ms^–1^. Consequently, DMS produces both small and large CCN sized particles throughout periods dominated by marine air masses.

**Table 3:** Median CCN Number Concentration Changes Due to Running The ADCHEM Model without DMS Emissions (woDMS), without Iodine Nucleation (woIodine), and without Anthroprogenic Emissions (woAnthro) Relative to The BaseCase Simulation During MP1, MP2, MP3, and CP1

	CCN change at *w* = 0.1 ms^–1^ (%)				CCN change at *w* = 1.0 ms^–1^ (%)			
Model Run	MP1	MP2	MP3	CP1	MP1	MP2	MP3	CP1
woDMS	–78.3	–81.5	–68.6	–6.0%	–60.8	–39.4	–51.2	–2.8
woIodine^a^	4.0	–2.4	4.2	–3.9	–2.1	–2.5	–0.3	–8.4
woAnthro	8.0	5.6	26.8	–14.2	–32.6	–30.3	–31.7	–23.3

a Iodine nucleation includes HIO_3_-HIO_2_ and HIO_3_-DMA. Anthroprogenic emissions comprise SO_2_, NO_*x*_, NH_3_, CO, BC, and AVOCs.

It is also evident that DMS alone constitutes a larger source of CCN sized particles at the SMEARII station compared to the effect from anthropogenic species. While running ADCHEM without anthropogenic emissions decreases the CCN number concentration by up to 33% at *w* = 1.0 ms^–1^, it also increases concentrations during periods dominated by marine air masses by up to 27% at *w* = 0.1 ms^–1^. This effect is likely caused by the anthropogenic contribution to NPF, resulting in more numerous but smaller particles that cannot activate into cloud droplets at low updraft velocities (low water vapor supersaturation).^20^

As discussed previously, iodine was found to have a minor impact on the accumulation mode particle concentration observed at the SMEARII station. Similarly, iodine has less of an effect on the CCN number concentration according to the cloud up-draft velocity model. Running ADCHEM without iodine derived NPF increases and decreases the amount of CCN particles by no more than ∼4% during periods of high marine air-mass impact (Table 3). Removing iodine NPF from the model does, however, decrease CCN productions by 8% at *w* = 1.0 m/s during CP1, where the air masses frequently arrived from coastal areas.

Based on the adiabatic cloud parcel model, it is clear that DMS influences the CCN particle number concentration and thus ultimately the local climate over the boreal forest. DMS initiates NPF over the oceans and land, forming aerosols in the nucleation and Atiken mode size range, which act as seed particles for the condensation of low volatile organic compounds when transported over forested land (Figure 4). This consequently leads to a significant increase in CCN particle number concentration over the boreal forest during periods of high marine air-mass impact. Iodine was found to have little influence on the CCN concentration locally at SMEARII, while still contributing to the nucleation and Aitken mode particle concentration during CP1. This effect corresponds with iodine-derived NPF originating from coastal emissions of iodine species, which do not have time to grow into the CCN size range upon reaching the SMEARII station. However, these particles may in turn reach CCN sizes further downwind from the station, ultimately affecting continental cloud processes and thus climate.

Besides DMS and iodine influencing the formation and growth of aerosol particles over the ocean and land in the present day atmosphere, it also appears likely that they may have had a significant impact on these aerosol processes in a preindustrial atmosphere. Even without anthropogenic precursors, DMS was found to contribute to relatively high concentrations of SO_2_ and SA at the SMEARII station, which in combination with natural emissions of NH_3_, DMA, iodine, and low volatile organics was found to drive the formation and growth of aerosol particles over the ocean and over land. Given the scale by which DMS and iodine is emitted globally from phytoplankton and open ocean, respectively, this process might also be relevant in other parts of the world. Furthermore, it may also have been the dominant driver in NPF and CCN production in the preindustrial atmosphere, and could become important in the future, as anthropogenic emissions of sulfur and NH_3_ continues to decrease while emissions of DMS and iodine continue to increase as a consequence of the global climate change.^8,72^

## Supporting Information Available

Figure S1 contains modeled and measured gas-phase concentrations of SO_2_, HIO_3_, and NO_*x*_, along with modeled gas-phase concentrations of OH, NH_3_, and DMA; Figure S2 illustrates the back trajectory heat maps for the full simulation period along with MP1, MP2, MP3, and CP1; Figure S3 demonstrates the method behind the air-mass origin analysis; Figure S4 contains the modeled size resolved chemical composition of SO_4_, NO_3_, Cl, NH_4_, Na, MSA, HIO_3_, DMA, SOA, and POA between 1 nm and 1 μm; Figure S5 illustrates the modeled and measured median particle number size distribution, including results from the *woDMS*, *woIodine*, and *woAnthro* sensitivity runs; Figure S6 contains modeled and measured particle numbers size distributions from Pallas, while Figure S7 contains similar results from Hyltemossa; Figure S8 illustrates the DMS, SO_2_, and OH gas-phase concentration along with the SA-NH_3_ nucleation rate along one of the HYSPLIT trajectories moving from the Norwegian Sea toward the SMEARII stationl Table S1 comprises COSMO*therm*-derived Henry’s law solubilities, while Table S2 contains COSMO*therm*-derived p*K*_a_ values (PDF)
