# Supplementary material for: Natural Marine Precursors Boost Continental New Particle Formation and Production of Cloud Condensation Nuclei
Source: Environ Sci Technol. 2024 Jun 13;58(25):10956–68. doi: 10.1021/acs.est.4c01891 (PMC11210206; doi:10.1021/acs.est.4c01891)
Supplement: Supplementary file 1 — es4c01891_si_001.pdf [file es4c01891_si_001.pdf]

Supporting information for: Natural marine precursors boost continental new particle formation and production of cloud condensation nuclei

Robin Wollesen de Jonge,* Carlton Xavier, Tinja Olenius, Jonas Elm, Carl Svenhag, Noora Hyttinen, Lars Nieradzik, Nina Sarnela, Adam Kristensson, Tuukka Petäjä, Mikael Ehn, and Pontus Roldin

* *Department of Physics, Lund University, Professorsgatan 1, Lund, SE-22363, Sweden*

E-mail: robin_wollesen.de_jonge@fysik.lu.se

List of Figures

S1 Additional gas-phase species . . . . P. S2
S2 Air-mass back trajectories . . . . P. S3
S3 Air-mass origin analysis . . . . P. S4
S4 Chemical composition . . . . P. S5
S5 Median particle number size distributions . . . . P. S6
S6 Particle size distributions (Pallas) . . . . P. S7
S7 Particle size distributions (Hyltemossa) . . . . P. S8
S8 Back-trajectory gas-phase concentrations . . . . P. S9

List of Tables

S1 COSMO*therm* Henry's law solubilities . . . . P. S10
S2 COSMO*therm* pK*a* . . . . P. S11

Additional Figures

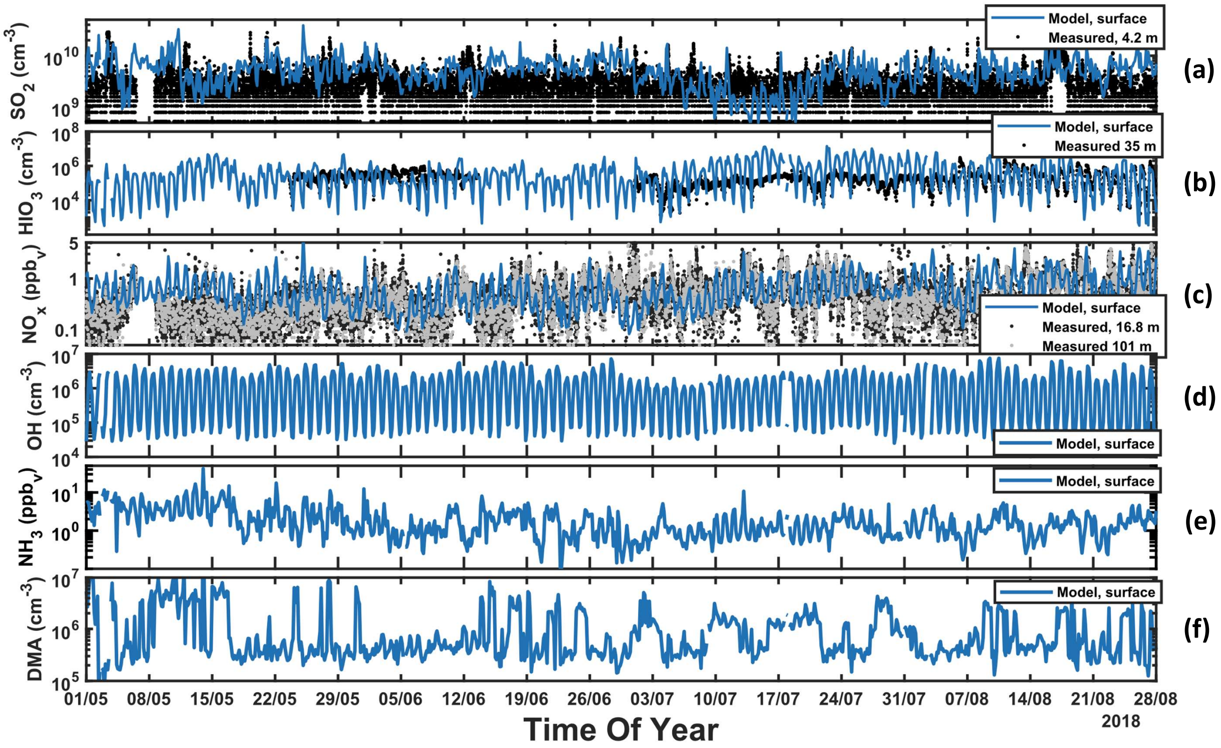

Figure S1: Modelled and measured long-term gas-phase concentrations of SO_2, HIO_3, NO_x, OH, NH_3 and DMA at the Station for Measuring Ecosystem-Atmosphere Relations II (SMEARII) between the 1th of May and 28th of August, 2018

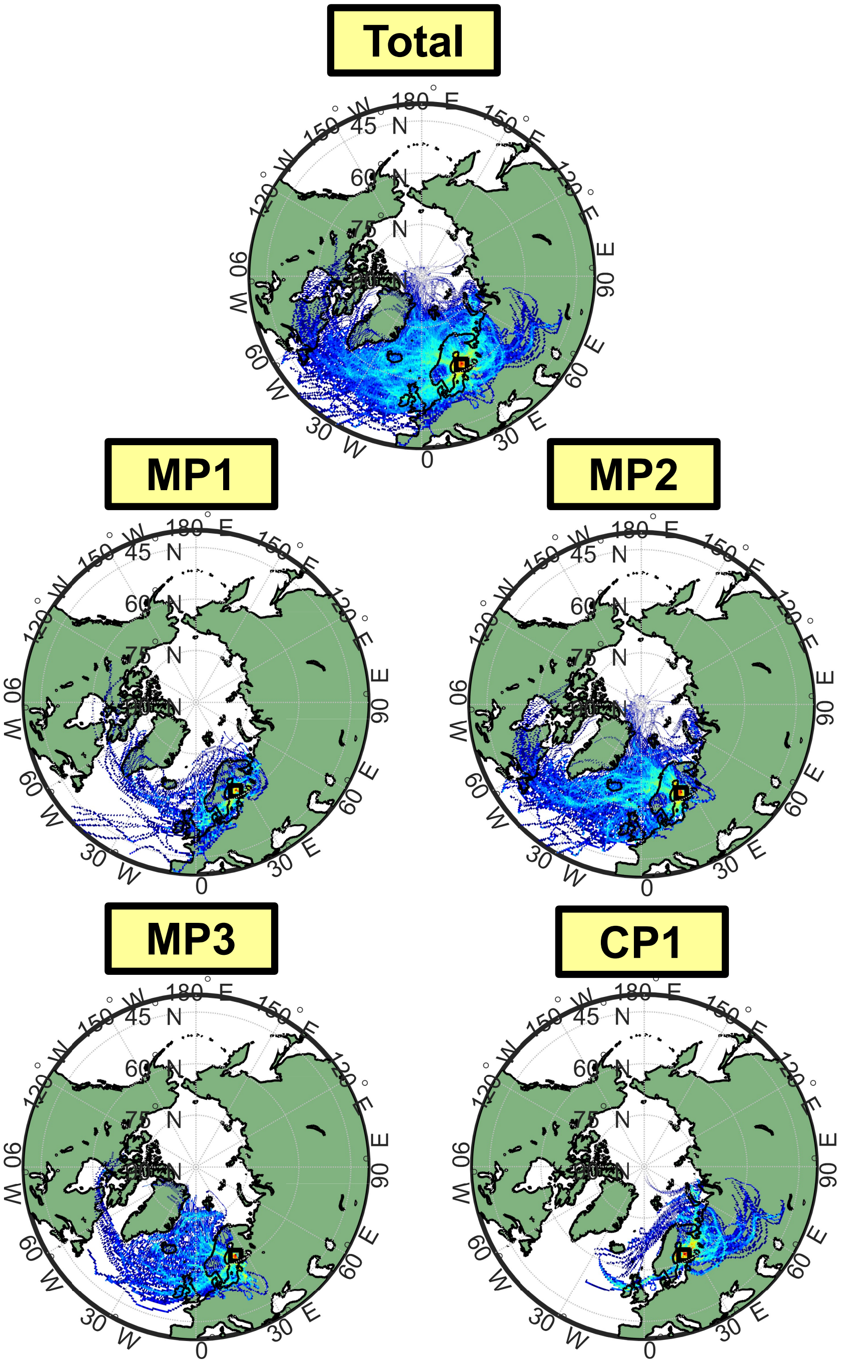

Figure S2: HYSPLIT air-mass back-trajectory heat maps. The complete period (Total) stretches between 01/05/18 - 28/08/18, while marine period period 1, 2, 3 (MP1, MP2, MP3) and continental period 1 (CP1) stretches between 17/05/18 - 10/06/18, 19/06/18 - 08/07/18, 05/08/18 - 28/08/18 and 10/07/18 - 03/08/18, respectively.

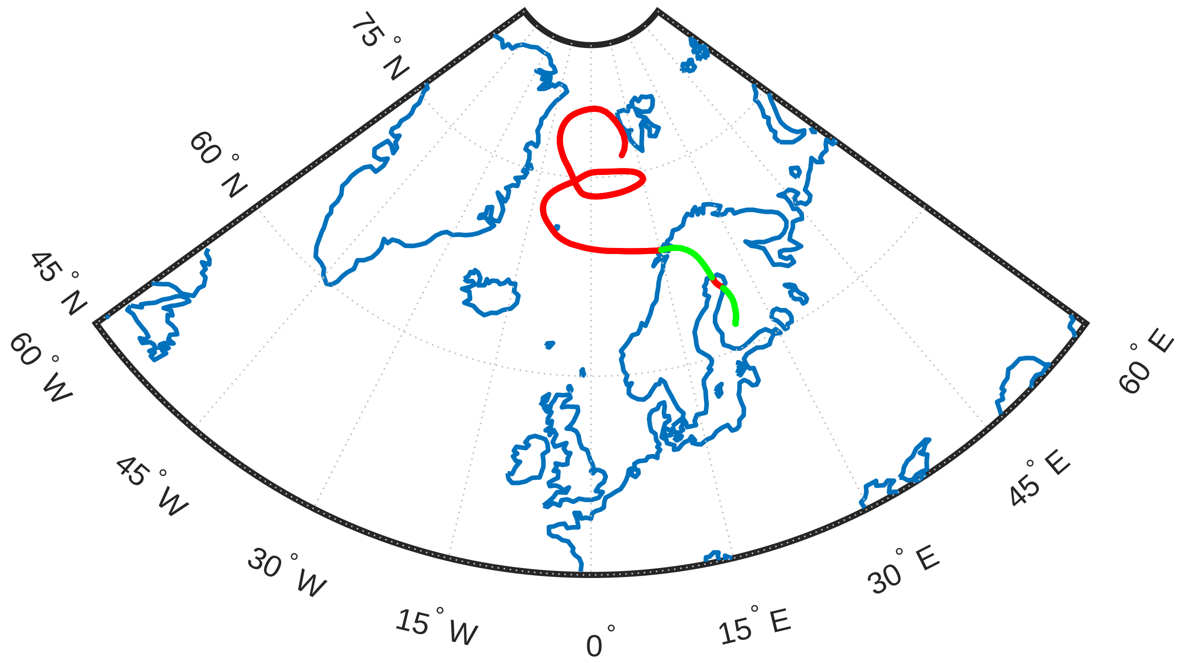

Figure S3: Principle behind the air-mass origin analysis. Each air-mass back trajectory is tested in terms of the time which it spent over the ocean before reaching the SMEARII station.

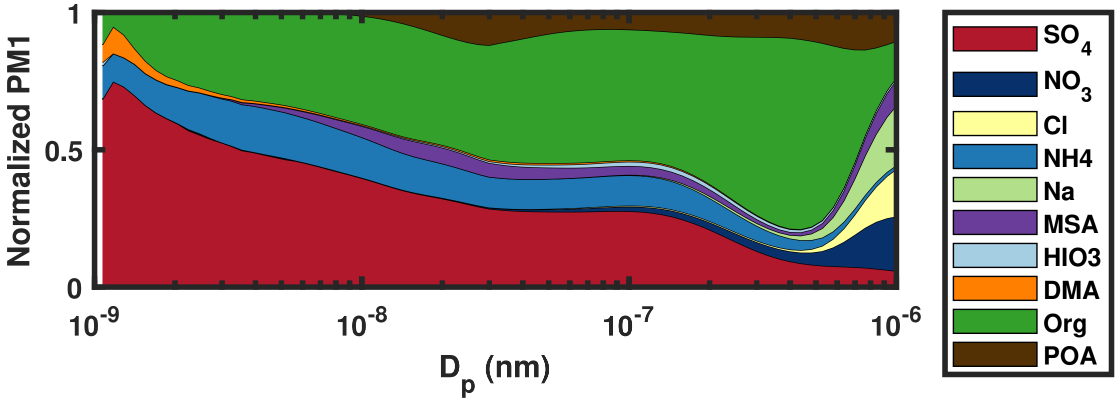

Figure S4: Modelled size resolved chemical composition of PM1 between the 1st of May and 28th of August, 2018.

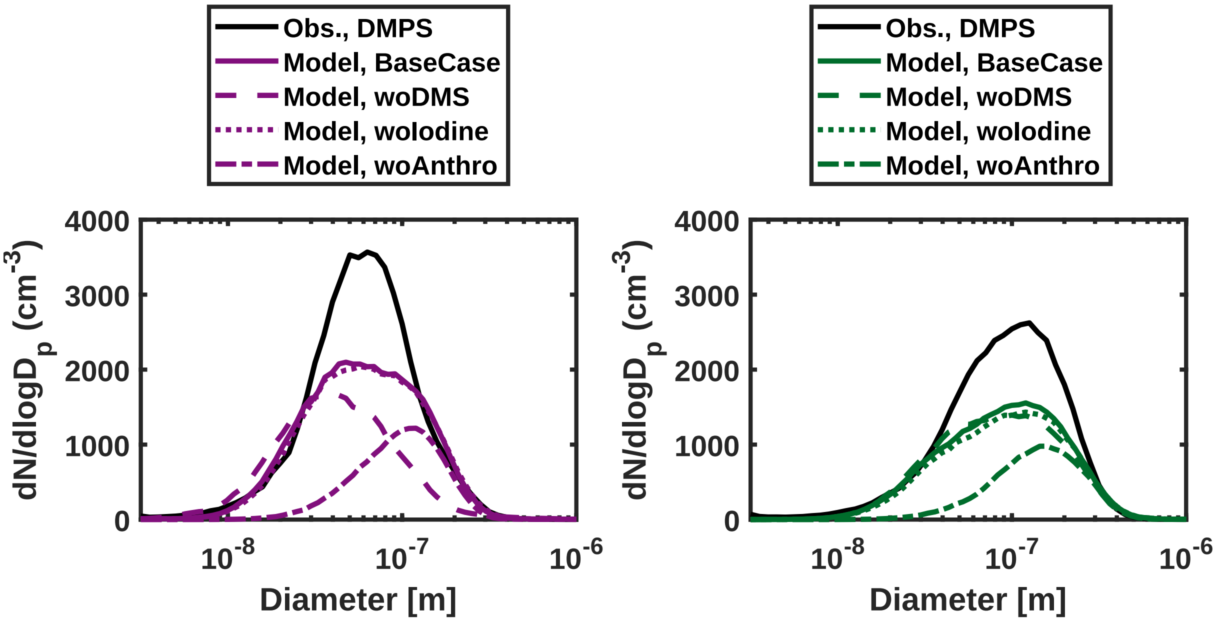

Figure S5: Measured and modelled median particle number size distributions at the Station for Measuring Ecosystem Atmosphere Relations II (SMEARII) between the 17th of May and 28th of August. The median size distributions are separated into periods of predominant marine air-mass impact (time spent over the ocean >50th prct., purple), and periods of predominant continental impact (time spent over the ocean <50th prct., green). The model results include data from the base case run (BaseCase), the without DMS emissions simulation (woDMS), the without iodine nucleation simulation (woIodine) and the without anthropogenic emissions simulation (woAnthro).

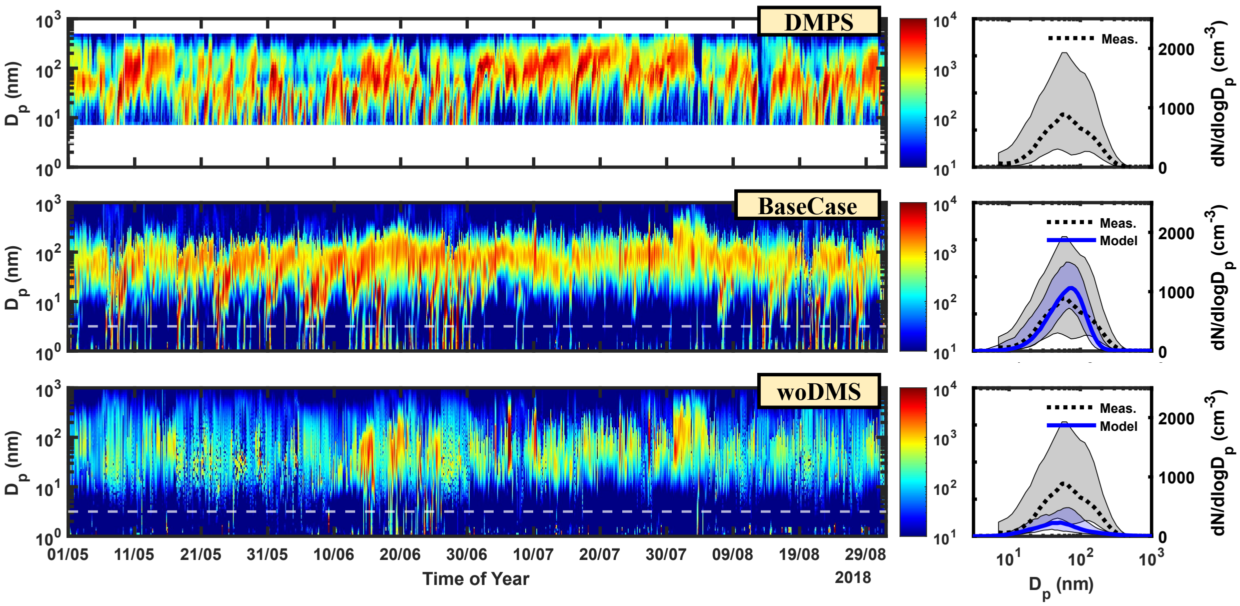

Figure S6: Measured and modelled time-dependant and median particle number size distributions at the Pallas research station between the 1st of May and 31st of August. The model results include data from the base case run (BaseCase) and the without DMS emissions simulation (woDMS). The shaded areas denote the measured and modelled data range within the 25th and 75th percentile

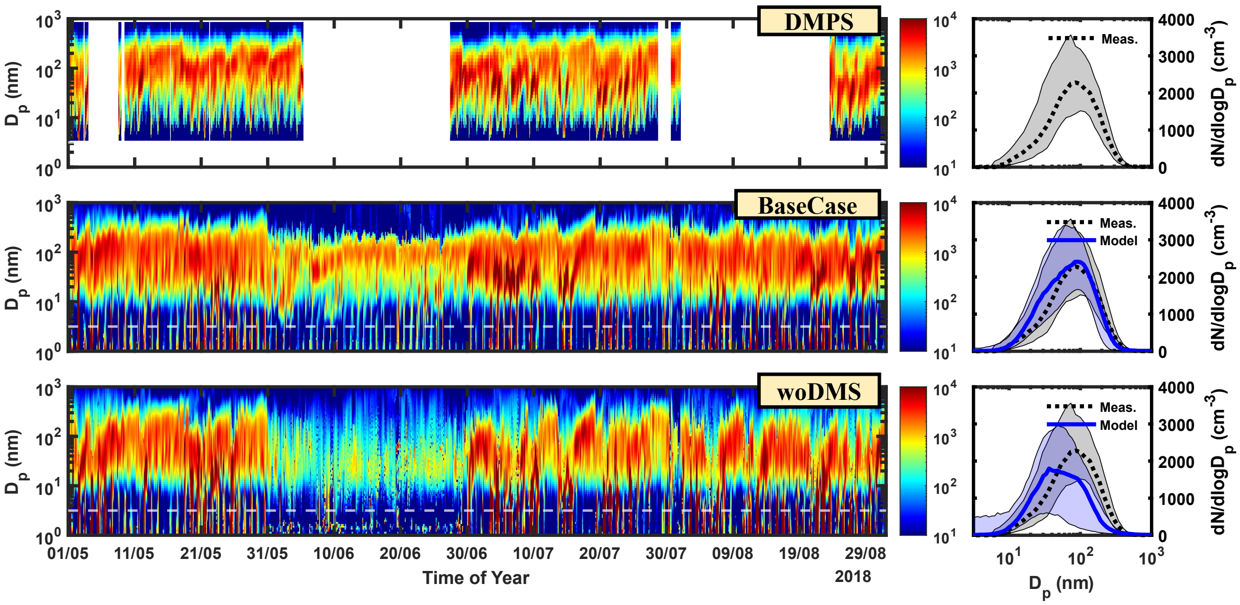

Figure S7: Measured and modelled time-dependant and median particle number size distributions at the Hyltemossa research station between the 1st of May and 31st of August. The model results include data from the base case run (BaseCase) and the without DMS emissions simulation (woDMS). The shaded areas denote the measured and modelled data range within the 25th and 75th percentile

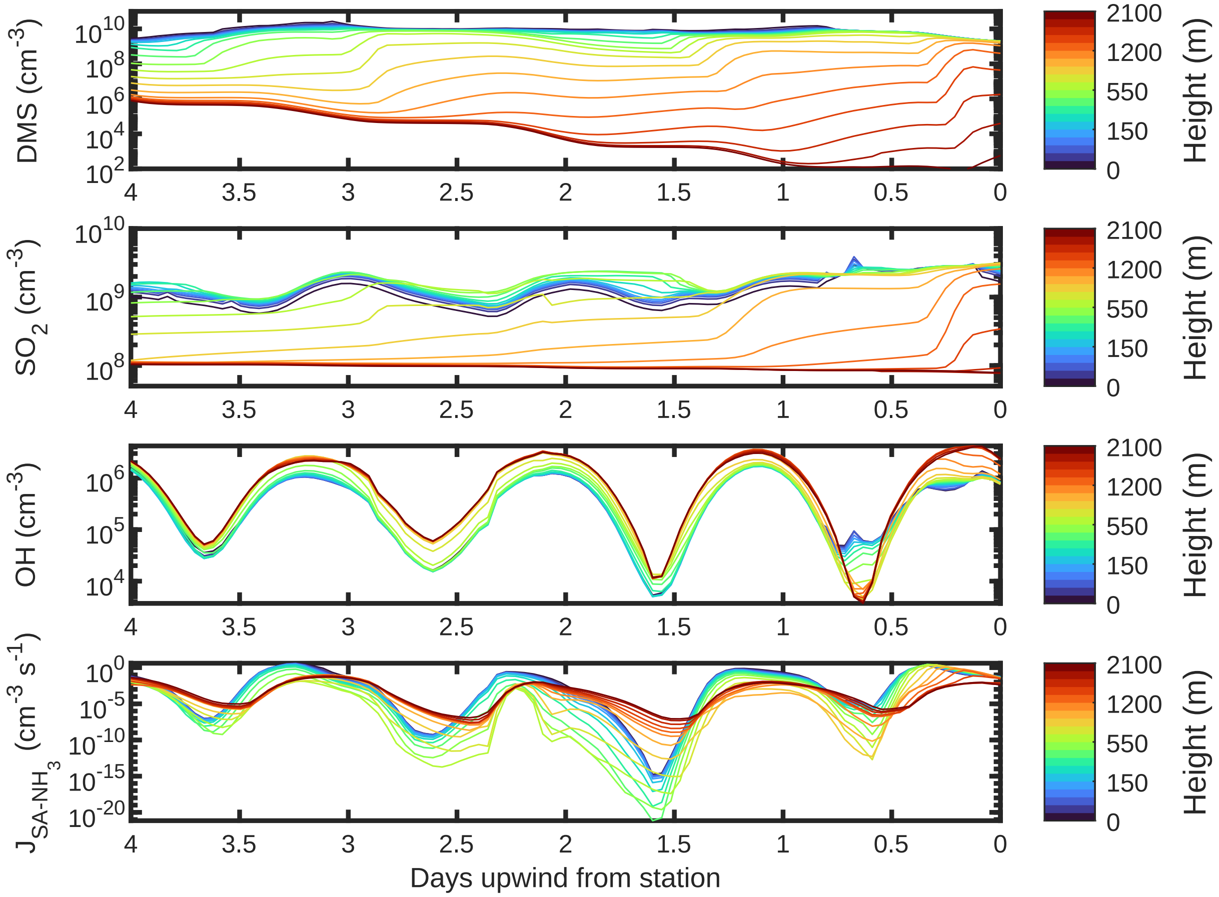

Figure S8: An example of the gas-phase concentrations of DMS, SO_2 and OH along with the SA-NH_3 NPF rates obtained from the ADCHEM model along a HYSPLIT back-trajectory moving from the Norwegian Sea towards the SMEARII station. All data is presented for the 20 model layers in ADCHEM spanning from 0 to 2100 meters.

Additional Tables

Table S1: COSMO*therm*-derived Henry's law solubilities H_{sol} in mol m^{-3} Pa^{-1}.

T(K)	MSIA	MSA	HIO_3	HIO_2
250	6.38E+06	4.61E+08	4.97E+09	2.929E+09
260	1.43E+06	9.72E+07	9.27E+08	5.138E+08
270	3.64E+05	2.34E+07	2.00E+08	1.044E+08
280	1.05E+05	6.34E+06	4.87E+07	2.419E+07
290	3.33E+04	1.91E+06	1.33E+07	6.303E+06
300	1.17E+04	6.29E+05	4.00E+06	1.825E+06

Table S2: COSMO*therm*-derived pK*a*.

T(K)	MSIA	MSA	HIO_3	HIO_2
250	2.27	-3.46	-2.67	4.30
260	2.34	-3.34	-2.57	4.36
270	2.41	-3.23	-2.47	4.42
280	2.48	-3.11	-2.37	4.48
290	2.56	-3.00	-2.27	4.54
300	2.63	-2.89	-2.17	4.61
